# Supplementary material for: Global green hydrogen-based steel opportunities surrounding high quality renewable energy and iron ore deposits
Source: Nat Commun. 2023 May 4;14:2578. doi: 10.1038/s41467-023-38123-2 (PMC10160127; doi:10.1038/s41467-023-38123-2)
Supplement: Supplementary file 1 — Supplementary Information [file 41467_2023_38123_MOESM1_ESM.pdf]

Global green hydrogen-based steel opportunities surrounding high quality renewable energy and iron ore deposits

-

Supplementary Information

S1. Supplementary Results

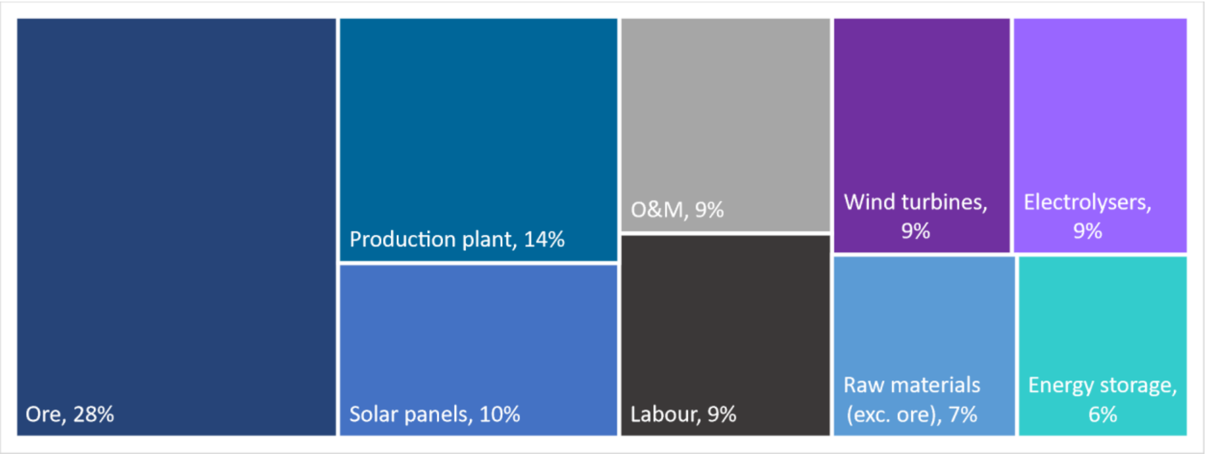

Figure S1 – Green hydrogen-based steel cost structure in 2050, average across all locations (0% scrap)

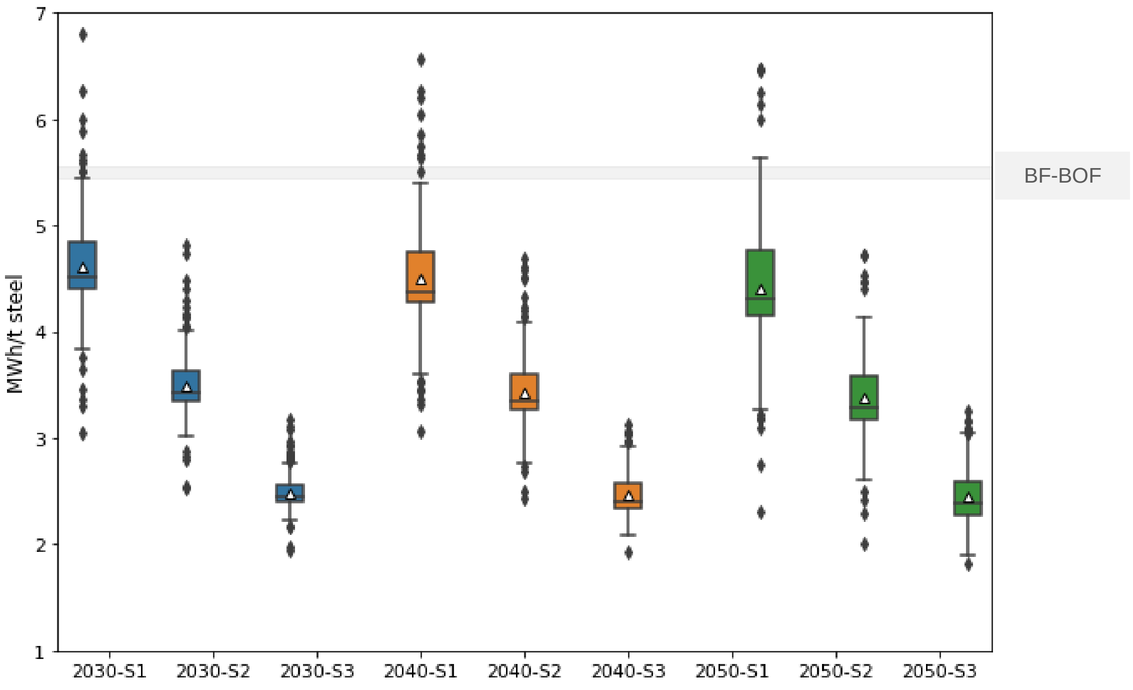

Figure S2 - Energy-intensity of iron and steel production for all installation years and scrap charges (case S1 = 0% scrap, S2 = 25% scrap, S3 = 50% scrap)

Table S1 – Renewable energy statistics for the 17 optimised countries

Country	Country code	mean hourly solar CF	median hourly solar CF	CoV hourly solar CF	mean monthly solar CF	median monthly solar CF	CoV monthly solar CF	mean hourly wind CF	median hourly wind CF	CoV hourly wind CF	mean monthly wind CF	median monthly wind CF	CoV monthly wind CF
**Australia**	AU	0.26	0.02	1.18	186.63	188.79	0.11	0.39	0.37	0.66	287.50	288.70	0.12
**Brazil**	BR	0.22	0.01	1.25	157.66	156.46	0.10	0.18	0.14	0.91	134.05	127.72	0.27
**Canada**	CA	0.15	0.00	1.54	111.48	116.72	0.31	0.33	0.27	0.86	239.83	235.26	0.16
**Chile**	CL	0.32	0.03	1.12	230.17	232.73	0.09	0.04	0.01	1.84	31.59	30.78	0.21
**China**	CN	0.22	0.01	1.29	163.46	164.52	0.13	0.33	0.28	0.77	241.61	245.79	0.20
**Guinea**	GN	0.19	0.00	1.30	141.97	136.94	0.12	0.11	0.07	1.08	77.28	69.48	0.29
**India**	IN	0.22	0.01	1.26	159.47	162.79	0.13	0.20	0.14	1.05	142.72	131.64	0.31
**Iran**	IR	0.30	0.03	1.13	217.12	220.20	0.08	0.24	0.18	0.85	176.83	176.41	0.20
**Kazakhstan**	KZ	0.20	0.02	1.27	147.29	158.47	0.25	0.41	0.39	0.53	296.82	297.66	0.12
**Mexico**	MX	0.23	0.01	1.25	171.19	172.45	0.11	0.13	0.09	1.34	97.14	95.08	0.20
**Peru**	PE	0.25	0.01	1.26	179.14	175.44	0.10	0.03	0.01	1.79	25.50	24.33	0.22
**Russia**	RU	0.14	0.01	1.50	100.89	98.85	0.42	0.28	0.25	0.72	207.46	205.20	0.17
**Sweden**	SE	0.13	0.00	1.68	91.34	85.82	0.52	0.26	0.22	0.78	193.31	186.61	0.20
**Turkey**	TR	0.25	0.03	1.22	184.58	185.10	0.18	0.19	0.10	1.18	135.10	139.16	0.24
**Ukraine**	UA	0.20	0.01	1.34	144.01	149.20	0.27	0.36	0.33	0.69	263.05	258.44	0.16
**United States**	US	0.21	0.01	1.33	154.42	158.91	0.18	0.32	0.28	0.75	236.07	236.22	0.16
**South Africa**	ZA	0.28	0.02	1.15	203.87	204.43	0.08	0.31	0.27	0.79	226.81	223.50	0.14

Table S2 – Flexibility characteristics of the steel production system (projects installed in 2050, no scrap charging)

Country	Total H_2 (t/t steel)	CGH_2 (t/t steel)	% CGH_2 of total H_2	HDRI (t/t steel)	CDRI (t/t steel)	% CDRI of total DRI	Electrolyser oversizing factor	EAF oversizing factor
**AU**	0.06	0.03	45%	1.00	0.26	21%	2.04	1.17
**BR**	0.07	0.05	65%	0.95	0.31	24%	3.00	1.32
**CA**	0.07	0.03	38%	0.95	0.30	24%	2.03	1.26
**CL**	0.09	0.06	70%	1.01	0.25	20%	2.88	1.21
**CN**	0.07	0.03	48%	0.99	0.27	21%	2.38	1.20
**GN**	0.08	0.06	75%	0.97	0.29	23%	3.67	1.35
**IN**	0.08	0.05	69%	0.97	0.28	22%	3.14	1.27
**IR**	0.08	0.05	66%	0.95	0.30	24%	2.88	1.29
**KZ**	0.06	0.01	23%	1.01	0.24	19%	1.32	1.13
**MX**	0.08	0.05	67%	1.00	0.26	20%	3.20	1.23
**PE**	0.08	0.06	75%	0.97	0.28	22%	3.18	1.34
**RU**	0.06	0.02	37%	0.97	0.28	23%	1.77	1.21
**SE**	0.07	0.03	39%	0.97	0.28	22%	1.81	1.19
**TR**	0.08	0.05	60%	0.94	0.32	25%	2.77	1.26
**UA**	0.06	0.02	34%	1.02	0.23	19%	1.69	1.15
**US**	0.06	0.03	43%	0.99	0.26	21%	2.01	1.20
**ZA**	0.07	0.04	54%	0.98	0.27	22%	2.44	1.22

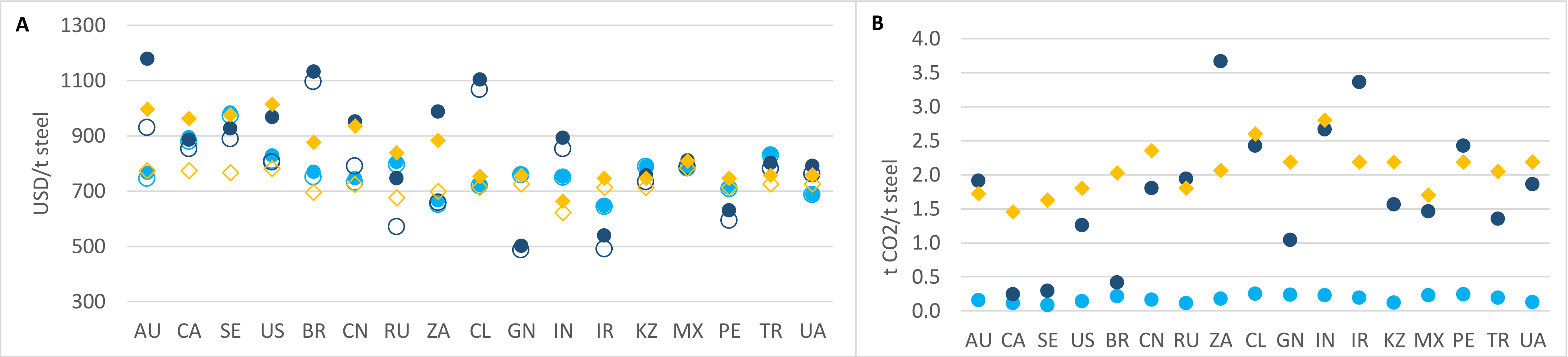

*Figure S3 – (a) Projected LCOS, with and without carbon taxes, and (b) CO_2 emission-intensity comparison across various steel production routes (**2030** project installation, no scrap): (i) H_2-DRI-EAF with islanded energy system, (ii) H_2-DRI-EAF powered by national grid and (iii) conventional BF-BOF route.*

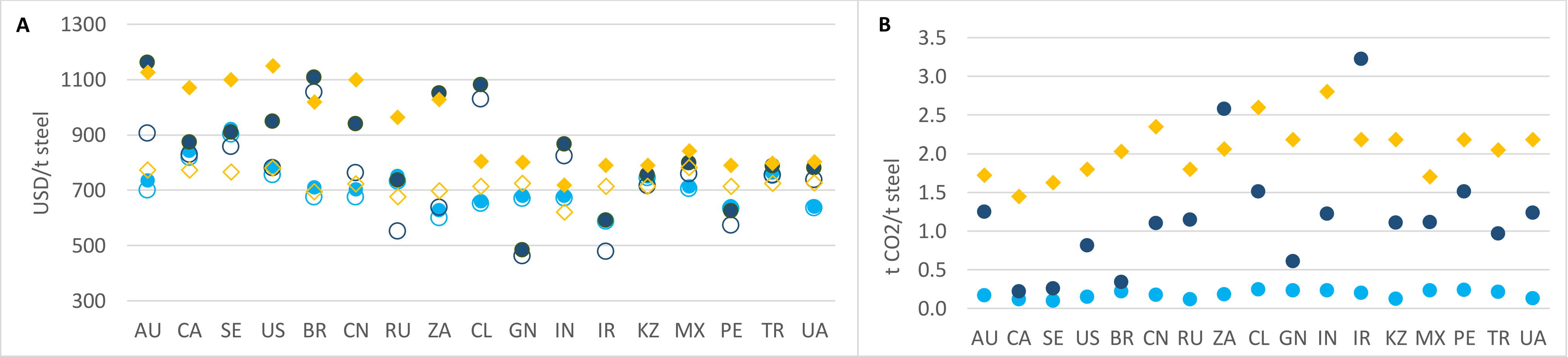

*Figure S4 – (a) Projected LCOS, with and without carbon taxes, and (b) CO_2 emission-intensity comparison across various steel production routes (**2040** project installation, no scrap): (i) H_2-DRI-EAF with islanded energy system, (ii) H_2-DRI-EAF powered by national grid and (iii) conventional BF-BOF route.*

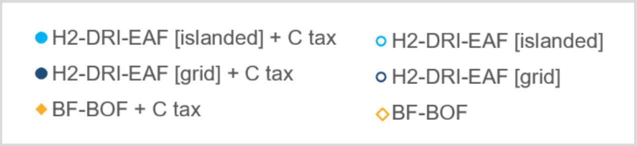

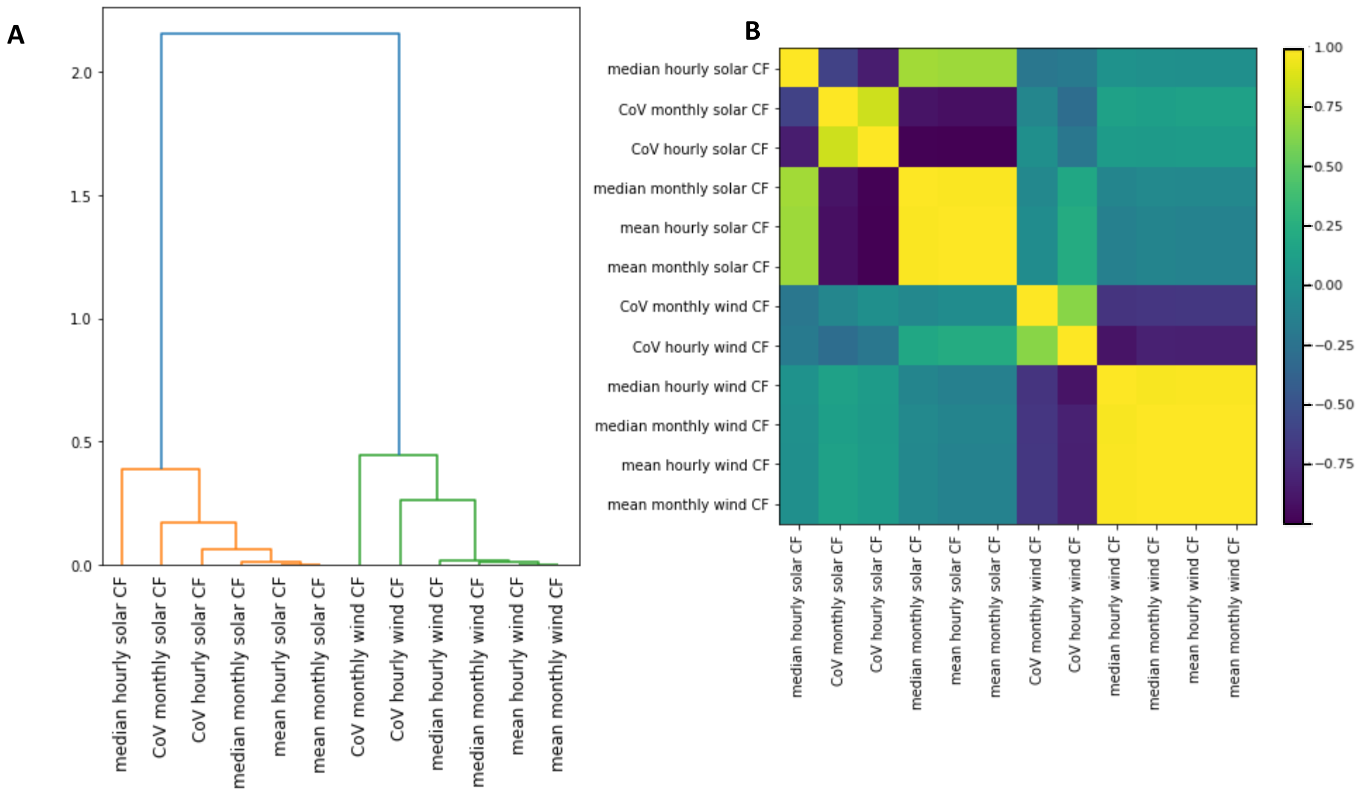

Figure S5 – (a) Multicollinearity analysis, and (b) Correlation matrix, of renewable energy statistics.

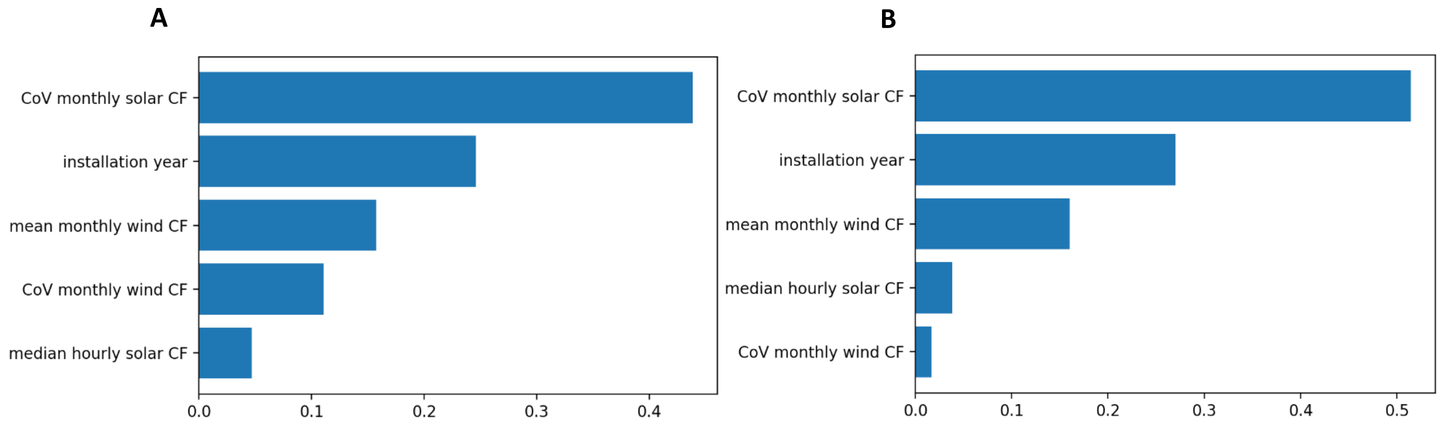

Figure S6 – Feature importance for machine learning models for (a) LCOS (exc. ore) and (b) RE cost.

Table S3 – Added ore and transport costs for LCOS at site, FOB and CFR economic assessments (2050, no scrap)

Country	LCOS, exc. ore	LCOS, inc. ore	LCOS FOB	LCOS CFR	Ore cost addition on top of LCOS at site	FOB cost increase from site costs (inc. ore)	CFR increase from FOB
**126.70**	553	679	687	720	23%	1%	5%
**125.95**	526	652	677	737	24%	4%	9%
**128.31**	651	779	801	855	20%	3%	7%
**132.69**	509	642	653	709	26%	2%	9%
**127.62**	468	595	625	639	27%	5%	2%
**109.75**	552	662	679	738	20%	3%	9%
**126.47**	537	664	671	702	24%	1%	5%
**109.50**	480	590	604	642	23%	3%	6%
**231.03**	471	702	745	805	49%	6%	8%
**124.75**	550	675	686	729	23%	2%	6%
**108.16**	531	639	651	706	20%	2%	8%
**107.15**	650	757	820	874	16%	8%	7%
**125.34**	473	598	614	659	27%	3%	7%
**120.28**	682	802	809	875	18%	1%	8%
**125.95**	579	705	719	767	22%	2%	7%
**126.10**	525	651	661	712	24%	2%	8%
**126.04**	599	725	779	825	21%	7%	6%

S2. Supplementary Discussion

S2.1 LCOS comparison to existing literature

In our previous study [1], the LCOS for localised production in Australia was cheaper: $526/t compared to $658/t in 2050 (no scrap charging). The cost difference was largely due to a simplified energy system in the former whereby a consistent diurnal solar profile was assumed and seasonal variations neglected (justifiable in the Pilbara region), as well as adjusted labour costs. The previous 100% solar RE system was superseded in this study by a 70% solar and 30% wind RE system, resulting from improvements over the simplified diurnal profile and wind turbine costs. Although the LCOE was cheaper in the previous study ($19/MWh, compared to $23/MWh now), the overall energy system costs (including hydrogen production and storage) were reduced in this work after optimisation ($241 down to $205/t steel). Wood, et al. [2] similarly looked at the Pilbara region in Australia for green steel production, estimating the production costs to be $677/t (removing water and transport, which this study excluded) with hydrogen modelled as an external input at $1.4/kg. This is very similar to the present study where Australia's insourced hydrogen costs were $1.74/kg and LCOS was $658/t in 2050.

A comparable variable-load H_2-DRI-EAF production system was explored by Pimm, et al. [3] for the UK, focusing on projects installed in 2040 with 50% scrap-charging, with results about $200/t steel cheaper than this study's global average. The key differences lie in cheaper labour rates, exclusion of continuous casting, and lower scrap prices: Pimm assumed a particularly advantageous scrap price ($164/t) compared to what is suggested by other sources [4,5] (approximately $265/t scrap) and the regionally differentiated scrap prices based on 2021 trade data that we used ($212-$624/t with global average of $422/t). Pimm also enabled dispatchable power in combination with storage to continuously supply H_2-DRI-EAF steelmaking, reducing process capacities of key flexible processes: electrolysers were oversized by factor of 1.8 (compared to global average of 2.4 for this study) whilst EAFs ran continuously (compared to global average oversizing factor of 1.24 for this study).

Vogl, et al. [4] explored the continuous-load H_2-DRI-EAF production system under theoretical optimum conditions, resulting in an LCOS range from $450/t steel (with $24/MWh electricity) to $778/t ($122/MWh electricity), where all 100% DRI systems (i.e. no scrap) required 274 MW electrolyser capacity. Comparatively, this study's cheapest steel was projected in Iran with LCOS of $535/t in 2050 with $20/MWh LCOE, however, this was only achievable through heavily oversized electrolysers, reaching 1000 MW capacity. Hence, favourable assumptions of constant production (and minimised infrastructure capacity) as well as cheap electricity tariffs accounted for the reduced LCOS projections.

S2.2 CGH_2 storage options

Required H_2 storage pressure, capacity, duration (of injection and withdrawal cycles) and responsiveness are of primary concern when selecting the storage medium. We modelled a steel pressure vessel, common in industrial applications for smaller scale and short-term applications for rapid energy storage utilisation, and available with net volume of 2.5-50m3 at pressure of 200-300 bar [6]. Geological storage (salt caverns, depleted gas reservoirs, aquifers, rock caverns) is the cheapest and most effective option for long-term, large-scale applications, however, location dependent. Salt cavern CGH_2 storage is already used commercially, with average storage capacity of 500,000 m^3 (economically inefficient below 150,000 m^3) [7] and the ability to provide seasonal energy

demand buffers. Depleted gas reservoirs are of much greater capacity but it is technically more difficult to store and extract CGH_2 from porous rocks, limiting the number of injection and withdrawal cycles per year [8]. A combination of CGH_2 storage in aboveground pressure vessels to provide a buffer for renewable energy's diurnal variation, and belowground geological storage for seasonal variations, would be desirable.

CGH_2 storage feasibility for the steel production system at-scale was assessed (refer to Supplementary Data for the full dataset). Depleted gas field storage is the most feasible option for H_2 storage in Australia, with enormous potential storage capacity estimated at 310 million tonnes, and in relatively close proximity to iron ore mines just off the NW coast in the Carnarvon Basin [9]. The scaled-up production system only requires 0.05% of this capacity. Following, nations with substantial natural gas operations will be ideal for H_2 storage (although also competing with CO2 storage sites). In comparison, Sweden's estimated underground capacity is almost negligible at 600 t (0.02 TWh, of which none is salt caverns) [10] which will not suffice the scaled-up system requirements (9850 t in 2050). Storage aboveground in 50m^3 storage vessels would also not be feasible at this scale, requiring nearly 14,000 units. The grid-connected system in Sweden may be necessary to secure renewable baseload (hydro) and mitigate H_2 storage altogether, which is the business model for Swedish green steel facilities in development.

S3. Supplementary Methods

Table S4 – Production sub-systems with breakdown of processes and storage

Sub-system	Processes	Storage
RE supply + battery storage	- Solar energy delivery<br>- Wind energy delivery<br>- Li-ion battery charging and discharging	Li-ion battery storage
Hydrogen production and storage	- Water electrolysis<br>- H_2 compression (200 bar)<br>- Fuel cell power production	CGH_2 storage
Ore mining and preparation	- Drilling and blasting<br>- Loading and hauling<br>- Crushing and screening<br>- Beneficiation<br>- Pelletising<br>- Stacking and reclaiming	Ore pellet storage*<br>Lump ore storage*
Ironmaking	- H_2 compression pre-DRI (2 bar)<br>- H_2 heating pre-DRI (to 900°C)<br>- Direct reduction of iron	Cold-DRI storage*
Steelmaking	- Cold-DRI heating (to 850°C)<br>- Electric arc furnace<br>- Continuous casting	Scrap storage*<br>Lime, alloys storage*<br>Steel storage*

**no energy or cost burdens associated with material storage*

Table S5 - Selected countries and key characteristics

Country	Alpha-2 code	No. regions	Economic status	Fe-content of extracted ore [11]
Australia	AU	2	Advanced	0.62
Brazil	BR	3	Major emerging	0.63
Canada	CA	4	Advanced	0.60
Chile	CL	1	Emerging market/developing economy	0.63
China	CN	6	Major emerging	0.61
Guinea	GN	1	Emerging market/developing economy	0.66
India	IN	3	Emerging market/developing economy	0.63
Iran	IR	1	Emerging market/developing economy	0.66
Kazakhstan	KZ	1	Emerging market/developing economy	0.20
Mexico	MX	2	Emerging market/developing economy	0.65
Peru	PE	1	Emerging market/developing economy	0.69
Russia	RU	8	Major emerging	0.71
Sweden	SE	1	Advanced	0.70
Turkey	TR	1	Emerging market/developing economy	0.56
Ukraine	UA	1	Emerging market/developing economy	0.63
United States	US	4	Advanced	0.63
South Africa	ZA	4	Major emerging	0.64

Table S6 – Key datasets for geospatial analysis

Data	Details	Sources
Solar and wind capacities	Hourly resource capacity based on 1kW capacity system and MERRA-2 climatic data. *Solar attributes: 1-axis rotation (azimuth), 10% system loss, 35° tilt, 180° azimuth. Wind attributes: 100m hub height, turbine model Vestas V90 2000.*	Renewables Ninja [12,13]
Iron ore deposit locations	Latitude and longitude of major global iron ore deposits.	Schulz and Briskey [14], published by U.S. Geological Survey
Iron ore reserve characteristics	Extracted ore iron ore and reserve statistics, including overall quantity and iron ore content, presented at national level.	U.S. Geological Survey [11] (and 5+ years of preceding annual publications)
Port locations	Latitude and longitude of global seaports, with classifications.	National Geospatial Intelligence Agency [15]

Table S7 – Mass loss rates in the ore preparation processes [16]:

Process	Mass losses
Crushing and screening	19%
Beneficiation	2.68% per ΔFe (%) in concentration
Pelletising	3%

Table S8 – Ore mining and preparation process energy demand

Process	Energy demand	Unit	Reference
Drilling and blasting	1.28	kWh/t crushed ore	Norgate & Haque (2010)
Loading and hauling	25.58	kWh/t crushed ore	Norgate & Haque (2010)
Crushing and screening	6.42	kWh/t crushed ore	Norgate & Haque (2010)
Communition (grinding + re-grinding)	27.36	kWh/t crushed ore	Palacios et al. (2019)
Concentration	0.85	kWh/t crushed ore-ΔFe(%)	Palacios et al. (2019)
Pelletising	208.28	kWh/t pellet	Lv et al. (2019)
Stacking and reclaiming	1.28	kWh/t pellet or lump	Norgate & Haque (2010)

Table S9 – Renewables technology cost and efficiency parameters

Parameter	Unit	2030	2040	2050	Reference
Solar PV panel cost	M$/MW	0.594	0.461	0.327	IRENA[17]
Wind turbine cost	M$/MW	1.088	0.962	0.835	IRENA[17]
Li-ion battery cost	M$/MWh	0.207	0.182	0.156	Cole and Frazier [18]
Electrolyser cost (low temp)	M$/MW	0.385	0.340	0.295	IEA[19]
Fuel cell cost (low temp)	M$/MW	0.457	0.267	0.218	Whiston, et al. [20]
Electrolyser efficiency (low temp, alkaline)	%	0.680	0.715	0.750	IEA [21]
FC efficiency (low temp)	%	0.600	0.620	0.640	IEA [22]

Battery, electrolyser, and fuel cells were assumed to have to be replaced twice in project lifetime, whereas all other technology pertained lifetimes that equalled or exceeded 20 years.

Table S10 – Resource consumption parameters

**ENERGY CONSUMPTION**	**Rate**	**Unit**	**Reference**
CGH_2 compression (200 bar)	2.87	MWh/t CGH_2	Devlin and Yang (2022)
Direct reduction of iron (H_2 feedstock)	48.13	kg H_2/t DRI	Devlin and Yang (2022)
H_2 heating pre-DRI	1606.10	MJ/t DRI	Devlin and Yang (2022)
H_2 compression pre-DRI	0.06	MWh/t DRI	Devlin and Yang (2022)
HBI briquette compression	0.01	MWh/t HBI	own calculations, based on data from [23]
HBI/CDRI heating	152.78	MWh/t HBI/CDRI	own calculations, based on data from [24]
Continuous casting	0.01	MWh/t steel	Devlin and Yang (2022)
EAF steelmaking			Kirschen, et al. [25]
- 0% scrap	0.75	MWh/t steel	
- 25% scrap	0.73	MWh/t steel	
- 50% scrap	0.71	MWh/t steel	
**MATERIAL CONSUMPTION**			
Ore demand			own calculations
- 0% scrap	1.73	dmt t/t steel	
- 25% scrap	1.24	dmt t/t steel	
- 50% scrap	0.79	dmt t/t steel	
Scrap demand			own calculations
- 0% scrap	0.00	t/t steel	
- 25% scrap	0.30	t/t steel	
- 50% scrap	0.57	t/t steel	
Lime demand			Kirschen, et al. [25]
- 0% scrap	50.00	kg/t LS	
- 25% scrap	49.50	kg/t LS	
- 50% scrap	49.00	kg/t LS	
Alloys	11.00	kg/t LS	Vogl et al. (2018)
Electrodes	2.00	kg/t LS	Vogl, et al. [4]
DRI metallisation rate (metallic Fe/total Fe)	94%		Kirschen, et al. [25]
EAF metal yield (mass of charged metal/mass of tapped steel)			
- 0% scrap	85%		own calculations
- 25% scrap	87%		own calculations
- 50% scrap	89%		own calculations
**LAND CONSUMPTION**			
Solar PV panels	0.02	km^2/MW	NREL [26]
Wind turbines	0.12	km^2/MW	NREL (2022)
**EMISSION-INTENSITY**			
Solar PV panels	48	g CO_2-e/kWh	Pehl, et al. [27]
Wind turbines	12	g CO_2-e/kWh	Pehl et al. (2017)

Table S11 – Economic parameters

Item	Value	Unit	References and notes
Real discount rate	8.00%	/yr	
Project lifetime	20	years	

OPEX

Scrap	265	$/t	Facchini, et al. [5]; Vogl et al. (2018)
Lime	121	$/t	Vogl et al. (2018)
Alloys	2,397	$/t	Vogl et al. (2018)
Carbon (graphite electrodes)	5,395	$/t	Vogl et al. (2018)
Labour - ironmaking (inc. H_2 production)	19	$/t DRI	OECD [28]; Vogl et al. (2018); Wood, et al. [2]
Labour - steelmaking (EAF, casting)	53	$/t steel	OECD (2021); Vogl et al. (2018); Wood et al. (2020)
Maintenance	2%	of CAPEX	Wood et al. (2020)

Infrastructure CAPEX

DRI shaft furnace	310	$/Mt rated DRI capacity	Vogl et al. (2018)
EAF unit - continuous mode	283	$/Mt rated steel capacity	Facchini et al. (2021); Steelonthenet.com [29]; Vogl et al. (2018)
Casting	0.945	$/t LS per hour (peak capacity)	Xylia, et al. [30]
CGH_2 plant (200 bar)	2.064	$/t H_2 per hour (peak capacity)	Abdin, et al. [31]
CGH_2 storage vessel (200 bar)	0.70	$/t CGH_2 stored (peak capacity)	Abdin et al. (2021)
H_2 compressor (2 bar)	8.4074x + 4.5351	$/Mt rated DRI capacity *where x = DRI production (Mtpa)*	Luyben [32]

Table S12 – Dry bulk freight rates

(a) Constant rates

Transportation cost item	Value	Unit	Reference
Rail freight	0.024	USD/t-km	van der Meulen, et al. [33]<br>Bureau of Infrastructure Transport and Regional Economics [34]
Panamax charter rate (exc. fuel)	0.0008	USD/t-km	BIMCO [35]
Panamax NH_3 consumption rate	0.22	t/nm	MAN Diesel and Turbo [36]
Port handling fee (load/unload)	7.00	USD/t	van der Meulen, et al. [33]<br>Abu Dhabi Ports [37]

(b) Time-variable rates

	2030	2040	2050		
NH_3 costs	713	570	460	$/t NH3	IRENA [38]
Fuel consumption (SOFC)	364	354	344	g/kWh	Devlin and Yang [1]

Table S13 – Industrial electricity tariffs

Country ID	Country	Industrial electricity tariffs, 2018 [39]	Data gaps filled by adjusted Cable.co.uk data*
AU	Australia	n/a	108.29
BR	Brazil	168.67	
CA	Canada	83.95	
CL	Chile	158.65	
CN	China	89.36	
GN	Guinea	n/a	20.62
IN	India	110.12	
IR	Iran	n/a	27.98
KZ	Kazakhstan	49.64	
MX	Mexico	89.53	
PE	Peru	n/a	50.34
RU	Russia	41.23	
SE	Sweden	87.2	
TR	Turkey	83.95	
UA	Ukraine	90.44	
US	United States	69.34	
ZA	South Africa	64.16	

**for the 4 countries where IEA data was not available, data for global household energy prices from Cable.co.uk [40] was adjusted using OECD average difference between household and industrial electricity tariffs (-37%) [41]*

Table S14 – BF-BOF emissions-intensity [42]

Country	t CO2/t steel
AU	1.72
BR	2.03
CA	1.45
CL	2.60
CN	2.35
GN	2.18
IN	2.80
IR	2.18
KZ	2.18
MX	1.70
PE	2.18
RU	1.80
SE	1.63
TR	2.05
UA	2.18
US	1.80
ZA	2.06

Italicised = economy-wide approximation

Table S15 – BF-BOF production costs [43]

Country	BF-BOF site costs (USD/t steel)	Assumptions
AU	773	*'advanced' economy average*
BR	694	
CA	773	*'advanced' economy average*
CL	714	*'developing' economy average*
CN	723	
GN	724	*no steel production - assume global average*
IN	621	
IR	714	*'developing' economy average*
KZ	714	*'developing' economy average*
MX	782	
PE	714	*'developing' economy average*
RU	676	
SE	766	*EU data from Medarac, et al. [44] (exc. credits) plus global avg. 2020-21 delta from Transition Zero [43]*
TR	725	
UA	726	
US	780	
ZA	698	*'major emerging' economy average*

Table S16 – Carbon prices [45]

C price (USD/t CO2) based on economy – net zero scenario			
Economy classification	*2030*	*2040*	*2050*
Advanced	130	205	250
Major emerging	90	160	200
Emerging market/developing economy	15	35	55

Table S17 – Regionally variable scrap prices based on trade volumes and economic value of flows under HS 720429 - Waste or scrap, of alloy steel, other than stainless [46]

Country	Import Qty (t)	Export Qty (t)	Trade position	Import price (USD/t, 2021 dollars)	Export price (USD/t, 2021 dollars)	Domestic price** (USD/t, 2020 dollars)
Australia	106,442	138,220	Exporter	491	444	424
Brazil	6,860	88,481	Exporter	1355	397	380
Canada	153,052	995,875	Exporter	256	494	472
Chile	6,763	21,699	Exporter	326	405	387
China	96,807	141	Importer	754	222	212
Guinea*	-	-	Importer	-	-	417
India	98,079	1,403	Importer	758	468	447
Iran*	-	-	Importer	-	-	417
Kazakhstan*	-	-	Importer	-	-	417
Mexico	94,772	234,685	Exporter	375	413	395
Peru	109,831	19,028	Importer	497	453	433
Russia	410,136	9,300	Importer	358	654	624
South Africa*	15,105	-	Exporter	163	-	417
Sweden	14,772	68,709	Exporter	1126	372	355
Turkey	125,741	11,102	Importer	427	1093	408
Ukraine	459	91	Importer	759	559	534
United States	696,763	903,352	Exporter	464	455	434

**assumed average medium quality scrap price due data unavailability*
***assumed export market drives the domestic price, excluding Turkey, the largest global scrap steel importer*

Table S18 – Regionally variable steelworker wages

	Average wage (before tax)	Steelworker wage (before tax)		Personal Income Tax Rate (highest)	Steelworker hourly rate, inc. tax
Source	[47]	30% increase above average [2]		[48]	
Unit	$/year	$/year	$/hr	% of income	$/hr
AU	53620	69706	36.31	45%	52.64
BR	7820	10166	5.29	28%	6.75
CA	43540	56602	29.48	33%	39.21
CL	12990	16887	8.80	40%	12.31
CN	10520	13676	7.12	45%	10.33
GN	960	1248	0.65	40%	0.91
IN	1890	2457	1.28	43%	1.83
IR	3290	4277	2.23	35%	3.01

KZ	8710	11323	5.90	10%	6.49
MX	8750	11375	5.92	35%	8.00
NZ	41480	53924	28.09	39%	39.04
PE	6000	7800	4.06	30%	5.28
RU	10740	13962	7.27	13%	8.22
ZA	6090	7917	4.12	45%	5.98
SE	54740	71162	37.06	52%	56.34
TR	9070	11791	6.14	40%	8.60
UA	3570	4641	2.42	18%	2.85
US	64650	84045	43.77	37%	59.97

Table S19 – Labour intensities

Process	Labour intensity	Source
Green H2 production	2 hours/kW installed electrolysers	Own calculations using data from Leguijt, et al. [49], considering labour demand for RE generation and electrolysis parts of value chain.
DR ironmaking	0.22 hours/t DRI	Own calculations using data from Wood, et al. [2].
EAF steelmaking	0.49 hours/t steel	

Supplementary References

1 Devlin, A. & Yang, A. Regional supply chains for decarbonising steel: Energy efficiency and green premium mitigation. *Energy Conversion and Management* **254**, 115268 (2022).

2 Wood, T., Dundas, G. & Ha, J. Start with Steel. (2020).<https://grattan.edu.au/wp-content/uploads/2020/05/2020-06-Start-with-steel.pdf>.

3 Pimm, A. J., Cockerill, T. T. & Gale, W. F. Energy system requirements of fossil-free steelmaking using hydrogen direct reduction. *Journal of Cleaner Production* **312**, 127665 (2021).

4 Vogl, V., Åhman, M. & Nilsson, L. J. Assessment of hydrogen direct reduction for fossil-free steelmaking. *Journal of Cleaner Production* **203**, 736-745 (2018).

5 Facchini, F., Mossa, G., Mummolo, G. & Vitti, M. An Economic Model to Assess Profitable Scenarios of EAF-Based Steelmaking Plants under Uncertain Conditions. *Energies* **14** (2021).

6 Elberry, A. M., Thakur, J., Santasalo-Aarnio, A. & Larmi, M. Large-scale compressed hydrogen storage as part of renewable electricity storage systems. *International Journal of Hydrogen Energy* **46**, 15671-15690 (2021).

7 Wang, Y., Kowal, J., Leuthold, M. & Sauer, D. U. Storage System of Renewable Energy Generated Hydrogen for Chemical Industry. *Energy Procedia* **29**, 657-667 (2012).

8 Scafidi, J. *Hydrogen Storage in Depleted Gas Fields: Capacity and Performance* Doctor of Philosophy thesis, The University of Edinburgh, (2021).

9 Ennis-King, J., Michael, K., Strand, J., Sander, R. & Green, C. Underground storage of Hydrogen: Mapping out the options for Australia. (CSIRO, 2021).<https://www.futurefuelscrc.com/wp-content/uploads/FutureFuelsCRC_UndergroundHydrogenStorage2021.pdf>.

10 Cihlar, J., Mavins, D. & van der Leun, K. Picturing the value of underground gas storage to the European hydrogen system. (Gas Infrastructure Europe, 2021).<https://www.gie.eu/wp-content/uploads/filr/3517/Picturing%20the%20value%20of%20gas%20storage%20to%20the%20European%20hydrogen%20system_FINAL_140621.pdf>.

11 U.S. Geological Survey. Mineral Commodity Summaries - Iron Ore. (2022).<https://pubs.usgs.gov/periodicals/mcs2022/mcs2022-iron-ore.pdf>.

12 Staffell, I. & Pfenninger, S. Using bias-corrected reanalysis to simulate current and future wind power output. *Energy* **114**, 1224-1239 (2016).

13 Pfenninger, S. & Staffell, I. Long-term patterns of European PV output using 30 years of validated hourly reanalysis and satellite data. *Energy* **114**, 1251-1265 (2016).

14 Schulz, K. & Briskey, J. *Major mineral deposits of the world: Open-File Report 2005-1294*, <https://mrdata.usgs.gov/major-deposits/> (2005).

15 National Geospatial Intelligence Agency. *World Port Index (pub 150)*, <https://msi.nga.mil/Publications/WPI> (2020).

16 Harvey, L. D. D. From Iron Ore to Crude Steel: Mass Flows Associated with Lump, Pellet, Sinter and Scrap Iron Inputs. *ISIJ International* **60**, 1159-1171 (2020).

17 International Renewable Energy Agency (IRENA). Future of Solar Photovoltaic: Deployment, investment, technology, grid integration and socio-economic aspects. (2019).<https://www.irena.org/-/media/Files/IRENA/Agency/Publication/2019/Nov/IRENA_Future_of_Solar_PV_2019.pdf>.

18 Cole, W. & Frazier, A. W. Cost Projections for Utility-Scale Battery Storage: 2020 Update. Report No. NREL/TP-6A20-75385, (Colorado, U.S., 2020).<https://www.nrel.gov/docs/fy20osti/75385.pdf>.

19 International Energy Agency (IEA). Net Zero by 2050. (IEA, Paris, 2021).<https://www.iea.org/reports/net-zero-by-2050>.

20 Whiston, M. M. *et al.* Meeting U.S. solid oxide fuel cell targets. *Joule* **3**, 2060-2065 (2019).

21 IEA. The Future of Hydrogen: Assumptions annex. (Paris, 2019).<https://www.iea.org/reports/the-future-of-hydrogen>.

22 IEA. Technology roadmap - hydrogen and fuel cells. (Paris, 2015).<https://www.iea.org/reports/technology-roadmap-hydrogen-and-fuel-cells>.
23 Lohmeier, L., Wollenberg, R. & Schröder, H.-W. Investigation into the Hot Briquetting of Fine-Grained Residual Materials from Iron and Steel Production. *steel research international* **91**, 2000237 (2020).
24 Midrex Technologies. DRI Products & Applications. (North Carolina, USA, 2018).<https://www.midrex.com/wp-content/uploads/MIdrexDRI_ProductsBrochure_4-12-18.pdf>.
25 Kirschen, M., Hay, T. & Echterhof, T. Process Improvements for Direct Reduced Iron Melting in the Electric Arc Furnace with Emphasis on Slag Operation. *Processes* **9** (2021).
26 National Renewable Energy Laboratory, U.S. Department of Energy. *Land Use by System Technology*, <https://www.nrel.gov/analysis/tech-size.html> (2022).
27 Pehl, M. *et al.* Understanding future emissions from low-carbon power systems by integration of life-cycle assessment and integrated energy modelling. *Nature Energy* **2**, 939-945 (2017).
28 OECD. *Average Annual Wages*, <https://stats.oecd.org/Index.aspx?QueryName=426&QueryType=View&Lang=en#> (2021).
29 Steelonthenet.com. *Capital Investment Costs - electric arc furnace*, <https://www.steelonthenet.com/capital-investment/eaf.html> (2022).
30 Xylia, M., Silveira, S., Duerinck, J. & Meinke-Hubeny, F. Weighing regional scrap availability in global pathways for steel production processes. *Energy Efficiency* **11**, 1135-1159 (2018).
31 Abdin, Z., Tang, C., Liu, Y. & Catchpole, K. Large-scale stationary hydrogen storage via liquid organic hydrogen carriers. *iScience* **24**, 102966 (2021).
32 Luyben, W. L. Capital cost of compressors for conceptual design. *Chemical Engineering and Processing - Process Intensification* **126**, 206-209 (2018).
33 van der Meulen, S. *et al.* Cost Figures for Freight Transport – final report. (Netherlands Institute for Transport Policy Analysis (KiM), 2020).
34 Bureau of Infrastructure Transport and Regional Economics. Freight Rates in Australia. (Canberra, 2017).
35 BIMCO. *Dry bulk - profits surge to multi-year highs as pandemic related demand and disruptions linger*, <https://www.bimco.org/news/market_analysis/2021/20210903-dry-bulk---profits-surge-to-multi-year-highs-as-pandemic-related-demand-and-disruptions-linger> (2021).
36 MAN Diesel and Turbo. Propulsion Trends in Bulk Carriers. (Denmark, 2014).<https://www.mandieselturbo.com/docs/default-source/shopwaredocumentsarchive/propulsion-trends-in-bulk-carriers.pdf?sfvrsn=0>.
37 Abu Dhabi Ports. Tariff of General Cargo Charges - Main Ports 2021. (2021).
38 IRENA. Innovation Outlook: Renewable Ammonia. (2022).<https://www.irena.org/publications/2022/May/Innovation-Outlook-Renewable-Ammonia>.
39 IEA. *World Energy Prices*, <https://doi.org/10.5257/iea/wep/2020> (2020).
40 Cable.co.uk. *Global energy pricing*, <https://www.cable.co.uk/energy/worldwide-pricing/> (2021).
41 IEA. Energy Prices and Taxes for OECD Countries. (Paris, 2020).<https://www.oecd-ilibrary.org/docserver/dbf6150b-en.pdf?expires=1660132551&id=id&accname=ocid57003439&checksum=24583519357C5E6A029496F671788BAE>.
42 Hasanbeigi, A. & Springer, C. How Clean is the U.S. Steel Industry? An International Benchmarking of Energy and CO2 Intensities. (Global Efficiency Intelligence, 2019).<https://www.belfercenter.org/sites/default/files/files/publication/how-clean-is-the-us-steel-industry-nv.pdf>.

43 Transition Zero. *Global Steel Cost Tracker*, <https://www.transitionzero.org/blog/stranded-assets-carbon-pricing-risk-steel> (2022).
44 Medarac, H., Moya, J. A. & Somers, J. Production costs from iron and steel industry in the EU and third countries. (European Commission, Joint Research Centre,, Netherlands, 2020).<https://www.eurofer.eu/assets/news/eu-technical-report-on-production-costs-from-the-iron-and-steel-industry-in-the-eu-and-third-countries/production_costs_from_the_iron_and_steel_industry_-_final_online.pdf>.
45 IEA. World Energy Model Documentation. (2021).<https://iea.blob.core.windows.net/assets/932ea201-0972-4231-8d81-356300e9fc43/WEM_Documentation_WEO2021.pdf>.
46 UNComtrade. *UN Comtrade Database*, <https://comtrade.un.org/data/> (2021).
47 World Bank Group. *GNI per capita, Atlas method (current US$)*, <https://data.worldbank.org/indicator/NY.GNP.PCAP.CD> (2022).
48 PwC. *Personal income tax (PIT) rates*, <https://taxsummaries.pwc.com/quick-charts/personal-income-tax-pit-rates> (2023).
49 Leguijt, C., van den Toorn, E., Bachaus, A. & Jongsma, C. Jobs from investment in green hydrogen. (CE Delft, 2021).<https://cedelft.eu/wp-content/uploads/sites/2/2021/04/CE_Delft_200427_Jobs_from_investment_in_green_hydrogen_Def.pdf>.
